# Physiological Responses of *Zostera marina* and *Cymodocea nodosa* to Light-Limitation Stress

**DOI:** 10.1371/journal.pone.0081058

**Published:** 2013-11-28

**Authors:** João Silva, Isabel Barrote, Monya M. Costa, Sílvia Albano, Rui Santos

**Affiliations:** 1 CCMAR - Centro de Ciências do Mar, Faro, Portugal; 2 CECTA - Centro de Estudos em Ciências e Tecnologias Agrárias, Universidade do Algarve, Faro, Portugal; Mount Allison University, Canada

## Abstract

The effects of light-limitation stress were investigated in natural stands of the seagrasses *Zostera marina* and *Cymodocea nodosa* in Ria Formosa coastal lagoon, southern Portugal. Three levels of light attenuation were imposed for 3 weeks in two adjacent meadows (2–3 m depth), each dominated by one species. The response of photosynthesis to light was determined with oxygen electrodes. Chlorophylls and carotenoids were determined by high-pressure liquid chromatography (HPLC). Soluble protein, carbohydrates, malondialdehyde and phenol contents were also analysed. Both species showed evident signs of photoacclimation. Their maximum photosynthetic rates were significantly reduced with shading. Ratios between specific light harvesting carotenoids and the epoxidation state of xanthophyll cycle carotenoids revealed significantly higher light harvesting efficiency of *C. nodosa*, a competitive advantage in a low light environment. The contents of both soluble sugars and starch were considerably lower in *Z. marina* plants, particularly in the rhizomes, decreasing even further with shading. The different carbohydrate energy storage strategies found between the two species clearly favour *C. nodosa's* resilience to light deprivation, a condition enhanced by its intrinsic arrangement of the pigment pool. On the other hand, *Z. marina* revealed a lower tolerance to light reduction, mostly due to a less plastic arrangement of the pigment pool and lower carbohydrate storage. Our findings indicate that *Z. marina* is close to a light-mediated ecophysiological threshold in Ria Formosa.

## Introduction

Seagrasses are exhibiting important declines worldwide. These are generally attributed to man-induced disturbances that lead to eutrophication and siltation, which deteriorate the light environment for these plants [1], [2], [3]. However, the mechanistic processes of physiological decline that ultimately lead to seagrass losses are not yet clear, partially because seagrass light requirements are not well understood. Published literature addressing the effects of light on seagrass photosynthesis has been recently reviewed [4]. The authors concluded that, despite the several published reports on the effects of light reduction on seagrass photosynthesis, morphology, growth and survival, essential knowledge on the underlying physiological mechanisms of light harvesting and resource allocation is still missing. Added constraints preventing generic assumptions for seagrasses are the interspecific variety of ecological strategies, growth rates, morphological and photosynthetic plasticity, photoacclimation potential and energy management strategies.

Following light reduction events, physiological responses are the first to occur, preceding morphological changes and the eventual biomass loss [5]. However, the type of response of different seagrass species to light reduction and/or deprivation appears to be highly related to specific morphology and leaf turnover rates [4]. Whereas smaller and faster-growing species are able to increase their leaf length or even replace them by new leaves, more adapted to low-light conditions, larger and slow-growing species must rely more on their capacity to adjust the photosynthetic apparatus and regulate the processes of light acquisition and energy conversion and storage. Adjustments in the photosynthetic apparatus to low light usually involve an overall increase of total chlorophyll and a reduction of the chlorophyll *a*∶*b* ratio [6] as a form of increasing photosynthetic efficiency, while the mobilization of carbohydrate reserves, mostly from rhizomes, provides a carbon source necessary to sustain growth [7], [5]. Ultimately, dealing with light reduction is an energetic balance issue, as plants try to optimize light energy harvesting while making the best possible use of stored carbon energy. The result of this interplay dictates the relative success with which seagrass species deal with periods of light reduction.

Of critical importance to understanding the light harvesting mechanism and its photoacclimation potential is detailed knowledge of the photosynthetic pigment pool. Light harvesting systems are able to adjust their operating efficiency to the light environment, shifting from high efficiency when light is limiting to photosynthesis, to lower efficiency when there is too much light. The modulation of photosynthetic pigments and soluble protein contents, as well as their balance, are part of this process and contribute to attaining a proper equilibrium between the energy input and output. In addition to chlorophyll *a* and *b*, the antennae of light harvesting complexes of terrestrial plants contain neoxanthin, lutein epoxide plus lutein and violaxanthin. Whereas chlorophyll *a* and *b* are always the main light capturing antennae pigments, higher proportions of neoxanthin, lutein epoxide and violaxanthin have been associated with more efficient light-harvesting antennae, eventually becoming acclimated to shady environments, and less prone to switch to the photoprotective mode [8], [9], [10].

In seagrasses, however, the analysis of photosynthetic pigments has so far been restricted to the quantification of chlorophylls and total carotenoids, whereas detailed analysis of the photosynthetic carotenoid pool has been scarce. Exceptions are [11], where the authors screened extracts from four Mediterranean seagrass species to identify the presence of the major photosynthetic carotenoids, [12] with the description of the diel evolution of the xanthophyll cycle pigments in *Z. marina* and [13], analysing the depth dependence of several carotenoids in *Posidonia sinuosa*. Here we present a comprehensive quantification of the photosynthetic carotenoid pool of *Cymodocea nodosa* and *Zostera marina*, describing the occurrence of seven photosynthetic carotenoids, with diverse physiological functions, from light harvesting to photooxidation prevention. Assessing the responses of the photosynthetic carotenoid pool to light reduction opens the way to further research aiming a better understanding of how seagrasses respond to transient or permanent shifts in their light environment, allowing a more detailed insight of the photo-physiological processes underlying such responses.

The aim of this study was to compare the short-term physiological responses of *Z. marina* and *C. nodosa* to different levels of light reduction. Specific objectives were (i) to investigate the effects of light reduction on the photosynthetic activity, (ii) to evaluate changes in the composition of the photosynthetic pigments pools and (iii) to examine the dynamics of carbohydrate synthesis, allocation and use in the above- and below-ground tissues.

## Materials and Methods

### Experimental design

No specific permissions were required to conduct the field experiments nor to collect biological samples in Ria Formosa coastal lagoon, according to the current national legislation. This work did not involve any endangered or protected species. Cymodocea nodosa and Zostera marina co-occur in the shallow subtidal of Ria Formosa coastal lagoon (South Portugal, 37°N, 8° W). Three levels of light attenuation were imposed in situ on both Z. marina and C. nodosa growing in two adjacent meadows at 3 m depth on February 2011. Five square plots (1 m2) were established per treatment using metallic structures covered with PVC mesh to obtain 24, 40 and 75% light attenuation relatively to ambient photosynthetic active radiation, PAR. Ambient PAR at 3 m depth reached maximum mid-day values of ca. 300 µmol quanta m−2s−1, throughout the experiment duration. In the shaded plots, maximum PAR was 228, 180 and 75 µmol quanta m−2s−1 (respectively 24, 40 and 75% attenuation). The shade screens were cleaned every two days to prevent fouling. The shading treatments were imposed for 3 weeks, at the end of which plant samples were collected for photosynthetic measurements and biochemical analysis. Control plants were collected in the natural meadows close to the shaded plots. Plants for biochemical analysis were collected, brought under shade to the surface, immediately cleaned of epiphytes, separated into leaves, rhizomes and roots, dried from excess water with paper tissue and frozen in liquid nitrogen. Plants for photosynthetic measurements were kept under shade and immersed in seawater for transportation to the laboratory, where they were kept overnight in a growth chamber, set to emulate the in situ measured temperature (15°C).

### Light response curves

The response of seagrass photosynthesis to the shading treatments was evaluated through P-E curves, measured with an oxygen electrode system (DW3/CB1, Hansatech, Norfolk, UK). Actinic light was provided by a slide projector (Pradovit 150, Leica, Germany) equipped with a halogen lamp (Osram Xenophot 150 W). A series of neutral density filters mounted on slide frames were used to obtain different light intensities. For each P-E curve, two leaf segments (2nd–3rd youngest leaves) of each replicate (n = 5) of either Z. marina or C. nodosa were clipped and mounted vertically side by side inside the measuring chamber for an even exposure to the incident light. GF/F filtered seawater (35‰) was used in the reaction vessel. The incubation chamber was coupled to a magnetic stirrer, which provided water homogenisation. Water temperature was kept constant at 15°C, controlled by a thermostatic circulator (Raypa, Spain). For each replicate curve, 10 light levels were applied sequentially, increasing from 5 to 875 µmol quanta m−2s−1 (PAR). Between light level exposures, the water from the reaction vessel was replaced by new water from the same original stock, previously brought to the measuring temperature. This water renewal prevents both oxygen super-saturation in the reaction chamber, with potential inhibitory effects on photosynthesis, and also the occurrence of significant pH drifts [14]. Each light level was imposed for approximately 8 min, enough time to obtain a straight line in the oxygen recording system, assumed as steady-state photosynthesis. P-E curves were fitted with the model equation of Smith and Talling [15], [16].
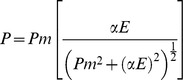
in which E is the irradiance, α is the ascending slope at limiting irradiances, and *P_m_* is the maximum photosynthetic rate. Curves were fitted iteratively using SigmaPlot 11.0 and the parameters *P_m_* and α as well as their standard error were estimated for a confidence interval of 95%. The saturation irradiance, *I_k_*, was calculated as the ratio between the estimated *P_m_* and α for each treatment, incorporating error propagation.

### Photosynthetic pigments

Photosynthetic pigments were extracted in 100 mg frozen leaf samples ground in liquid nitrogen in the presence of sodium ascorbate. Pigments where extracted with 5 mL 100% acetone buffered with CaCO_3_ [17]. The extracts were sequentially filtered with LS 5.0 µm membrane filters and hydrophobic PTFE 0.2 µm filters, and stored in the dark at −20°C until analysis. The extraction procedure took place under low light environment. Chlorophyll *a* (*Ca*) and *b* (*Cb*) were quantified by spectrophotometric absorbance reading, using the equations of Lichtenthaler and Buschmann [18]. Carotenoids were analysed in an isocratic High Performance Liquid Chromatography (HPLC), as described in [19] after [20]. HPLC calibration [20] was done using commercially available pigments (CaroteNature, Lupsingen, Switzerland). Liquid chromatography analysis was performed in an Alliance Waters 2695 separation module (Milford MA, USA), with a Waters 2996 photodiode array detector and a Waters Novapak C18 radial 8×100 mm compression column (4 µm particle size). 20 µL of extract were injected via an auto-sampler. During the injection period, extracts were maintained at 5°C and the column was kept at a constant temperature of 24°C. All eluents were prepared with HPLC grade solvents (VWR Hipersolv Chromanorm), filtered and sonicated prior to use. Peak areas were monitored at 450 nm and concentrations were calculated based on peak areas obtained for standards at known concentrations, calculated based on absorbance measured in a Beckman-Coulter DU 650 spectrophotometer (Brea CA, USA). The xanthophyll cycle epoxidation state (EPS) was calculated based on violaxanthin (V) anteraxanthin (A) and zeaxanthin (Z) foliar concentrations as in [21]: EPS  =  (V+0.5A)/(V+A+Z).

### Soluble protein

Frozen leaf samples (150 mg each) were ground in 1.5 mL of protein extraction buffer (100 mM Potassium phosphate, pH 7.8, 1 mM DTT, 1 mM PMSF, 2% (v/v) Triton-X). The extract was centrifuged at 18000×g for 2 min at 4°C and the supernatant was collected. Soluble protein concentration was determined by a dye-binding assay (Coomassie Brilliant Blue G-250 dye) [22], against a Bovine Serum Albumin standard (BioRad).

### Non-structural carbohydrates

Freeze-dried samples of leaves and rhizomes (n = 5, 10 mg DW each) were ground to powder on a ball mill, extracted in ethanol at 80°C for 10 min. and centrifuged at 2000 g for 5 min. [23]. The supernatant was collected and the pellet was ressuspended in ethanol for additional extraction. This procedure was repeated a third time to allow full extraction of soluble sugars (glucose, sucrose and fructose). The supernatants from the three-step extraction were mixed together and the amount of soluble sugars was determined by a phenol-sulphuric assay [24] using glucose standards. For starch quantification, the pellet was washed in deionised water, centrifuged, ressuspended again in water (repeated three times) and autoclaved for 15 min. Starch was hydrolysed to glucose in the presence of an enzymatic complex (14 U/ml amyloglucosidase and 1000 U/mg α-amylase per sample) and determined as glucose equivalents following the phenol-sulphuric assay described above.

### Total phenols

Frozen leaf samples (*ca.* 200 mg fresh weight) were powdered in liquid nitrogen and total phenols were extracted and quantified as in [25], [26]. Extracts were suspended in 0.1 mol L^−1^ HCl and kept overnight at 4°C in the dark, under constant agitation. Following centrifugation, 0.25 N Folin-Ciocalteu reagent and 7.5% Na_2_CO_3_ were added to the supernatant. Absorbance was read at 724 nm in a Beckman Coulter DU-650 spectrophotometer (Brea CA, USA), against a blank sample. The assay results were expressed as chlorogenic acid equivalents.

### Malondialdehyde (MDA)

For MDA extraction, *ca.* 300 mg of frozen leaf samples were ground to powder in liquid nitrogen and suspended in 80% aqueous ethanol. After centrifugation the supernatant was added to a solution of 20% trichloroacetic acid (TCA) with 0.65% thiobarbituric acid (TBA) and 0.015% butylated hydroxytoluene (BHT). Two blanks were done either without TBA or with 80% ethanol instead of sample extract. All samples and blanks reaction mixtures were heated (90°C, 25 min), then cooled (ice bath, 15 min) and again centrifuged. Absorbances were read in the supernatants at 532 nm, 600 nm and 440 nm using a Beckman Coulter DU-650 spectrophotometer and MDA equivalents were calculated as in [27].

### Statistical analysis

All results are presented as mean values ± standard error of replicate samples (n = 5), except when noted differently. When not stated otherwise, one or two way ANOVAs were applied to test significant effects (p<0.05). Student-Newman-Keuls post-hoc method was used to reveal significant differences between individual means [28]. All data treatment and statistical analysis was performed using the SigmaStat/SigmaPlot (SPSS Inc., v.11) software package.

## Results

### Light response curves

**Figure 1:**
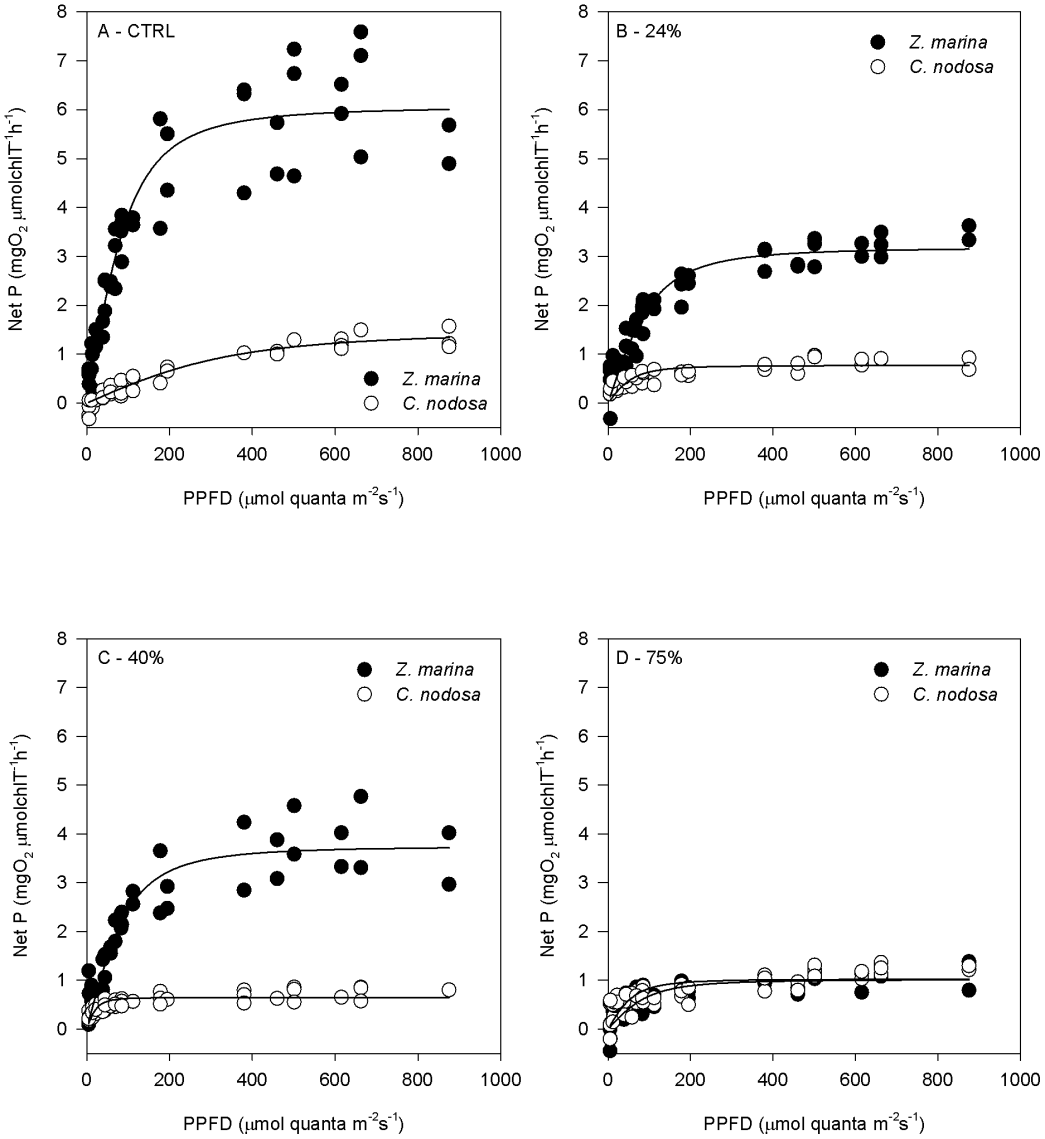
Light response curves of *Zostera marina* and *Cymodocea nodosa*. Plants submitted to shading treatments of 24, 40 and 75% of naturally available photosynthetically active radiation (CTRL). The model equation of Smith and Talling [15] [16] was adjusted to the observed points.

The maximum photosynthetic rates of both *Zostera marina* and *Cymodocea nodosa* were significantly reduced with the shading treatment (Fig. 1 and Table 1). In *Z. marina* plants, reductions in *P_m_* increased with the shading level, with significant differences from control to all the shading levels, with the 75% shading level displaying the lowest *P_m_*. In *C. nodosa*, all levels showed significant differences in *P_m_* relative to the control but not among them; the plants under 75% shading were the exception, with significantly higher *P_m_* than 24% and 40% shading levels. With the exception of the highest shading level, *Z. marina P_m_* rates were always significantly higher (three-fold or more) than those of *C. nodosa*. The ascending slope at limiting PPFDs (α) decreased in *Z. marina* from the control to all the shading levels, while in *C. nodosa* an opposite trend was observed, with all the shading levels displaying higher α values than control plants (Table 1). The saturation irradiance (*I_k_*) of *Z. marina* was not affected by shading, whereas in *C. nodosa* it decreased at least four-fold from the control to all shading levels, with no significant differences among these (Table 1).

**Table 1:** Photosynthetic parameters obtained from the adjustment of the model equation of Smith and Talling [15] [16] to the observed P-E data for *Zostera marina* and *Cymodocea nodosa*.

Shading treatment	*P_m_*	*α*	*I_k_*	r^2^
*Z. marina*				
CTRL	6.06* ^a^±0.19	0.0498* ^a^±0.0038	121.71±10.06	0.90
24%	3.18* ^c^±0.09	0.0253* ^b^±0.0017	125.66*±9.17	0.91
40%	3.75* ^b^±0.14	0.0321^b^±0.0029	116.74*±11.43	0.88
75%	1.01^d^±0.06	0.0091^c^±0.0013	111.51±17.28	0.76
*C. nodosa*				
CTRL	1.47^a^±0.09	0.004^b^±0.0003	397.68* ^a^±40.08	0.93
24%	0.77^c^±0.04	0.0113^b^±0.0016	68.34^b^±10.26	0.62
40%	0.65^c^±0.03	0.0236^a^±0.0039	27.49^b^±4.68	0.58
75%	1.03^b^±0.06	0.0142^ab^±0.0023	72.15^b^±12.40	0.50

*Zostera marina* and *Cymodocea nodosa* plants submitted to shading treatments of 24, 40 and 75% of naturally available photosynthetically active radiation (CTRL). Values are means ± se (n = 5, p<0.001). *P_m_* is the maximum photosynthetic rate (mg O_2_ µmol ChlT^−1^h^−1^), α is the ascending slope of the light response curves at limiting PPFDs (mg O_2_ µmol ChlT^−1^h^−1^ (µmol quanta m^−2^s^−1^)^−1^), *I_k_* is the saturation irradiance (µmol quanta m^−2^s^−1^) and r^2^ is the coefficient of determination of the model adjustment to the data. Different letters indicate significant differences between treatments,

*indicates differences between species (*p*<0.05).

### Photosynthetic pigments

**Figure 2:**
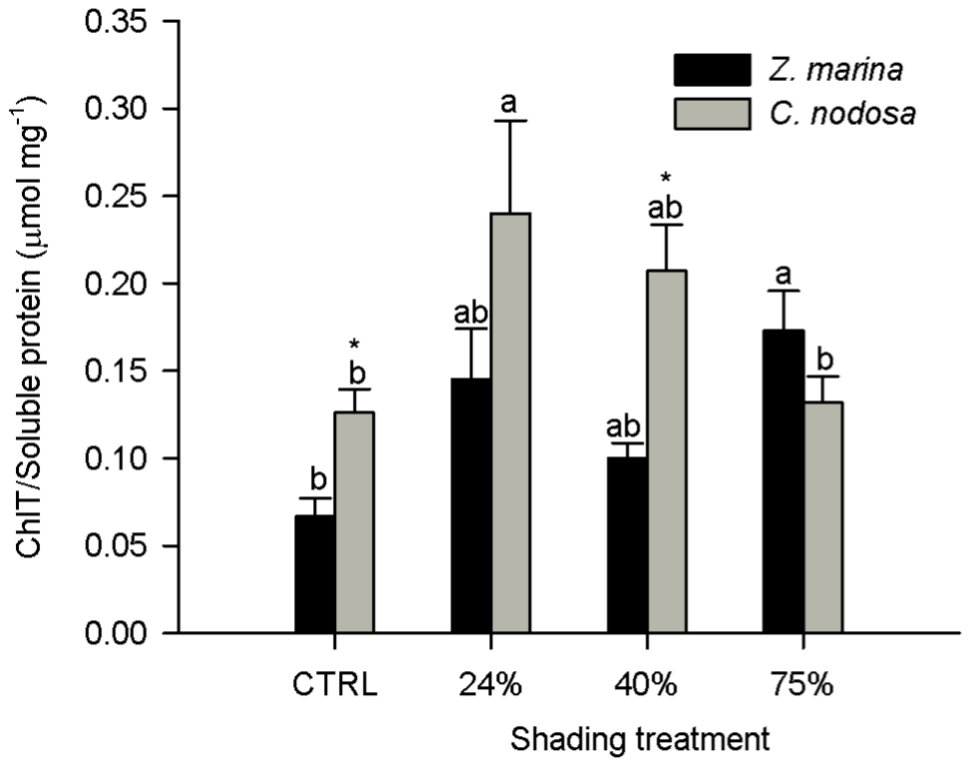
Total chlorophyll to soluble protein ratio (ChlT/Protein) in leaves of *Zostera marina* and *Cymodocea nodosa*. Plants submitted to shading treatments of 24, 40 and 75% of naturally available photosynthetically active radiation (CTRL). Different letters indicate significant differences between treatments, * indicates differences between species (n = 5, *p*<0.05).

The total chlorophyll/soluble protein ratio in Z. marina leaves increased with shading (Fig. 2), up to 2.6 fold at the 75% shading level. In C. nodosa, the ratio peaked at the 24% level, the only one with a significant difference from the control. The ChlT/protein ratio was higher in C. nodosa than in Z. marina, with significant differences observed both in control and 40% shading levels.

**Figure 3:**
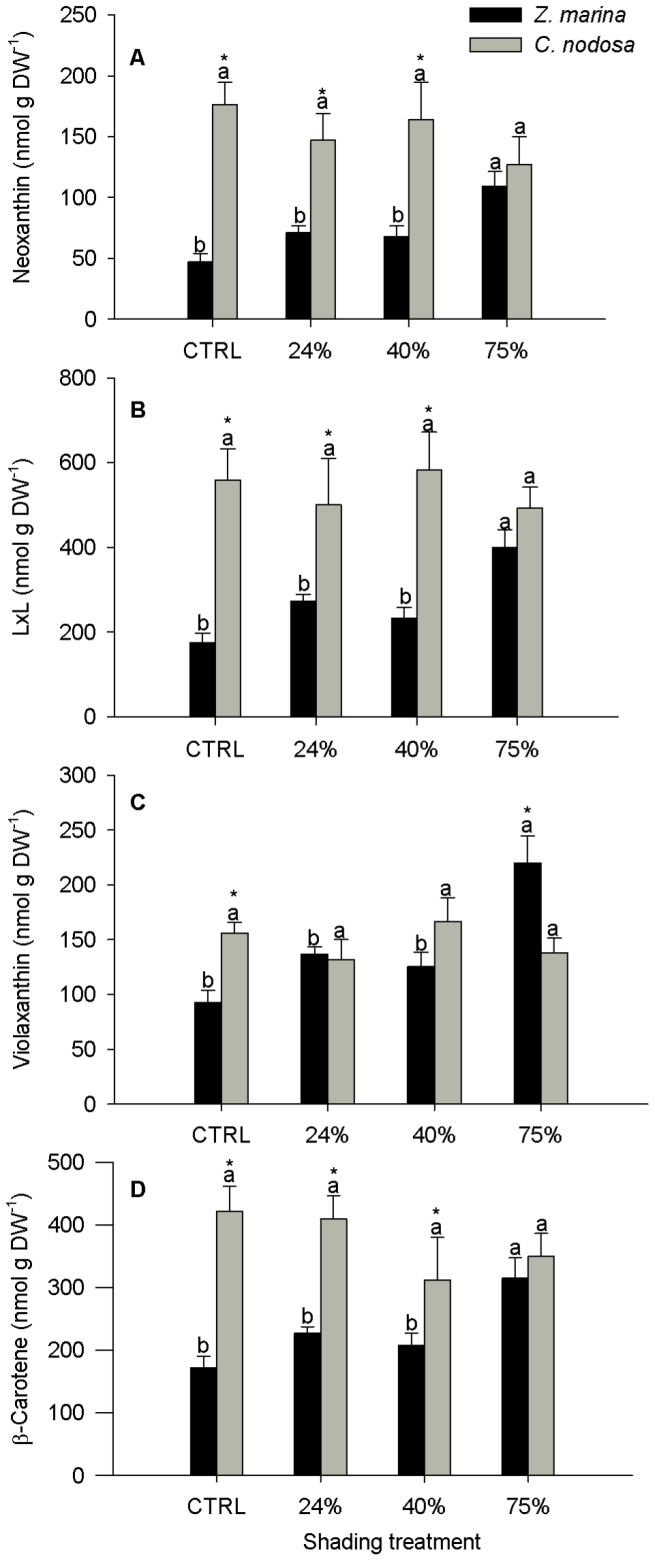
Foliar content of photosynthetic pigments in *Zostera marina* and *Cymodocea nodosa*. Plants submitted to shading treatments of 24, 40 and 75% of naturally available photosynthetically active radiation (CTRL). A- Neoxantin, B - Lutein plus Lutein epoxide (LXL), C -Violaxanthin, D –β-Carotene. Different letters indicate significant differences between treatments, * indicates differences between species (n = 5, *p*<0.05).

In both species, the photosynthetic carotenoids neoxanthin, lutein epoxide + lutein (LxL), violaxanthin and β –carotene presented identical patterns of response to shading (Fig. 3 A–D). In C. nodosa, none of these pigments showed a significant difference among treatments, whereas in Z. marina their concentration increased only at the 75% shading level, by a factor of 2.3 in neoxanthin, LxL and violaxanthin and 1.8 in β – Carotene.

**Figure 4:**
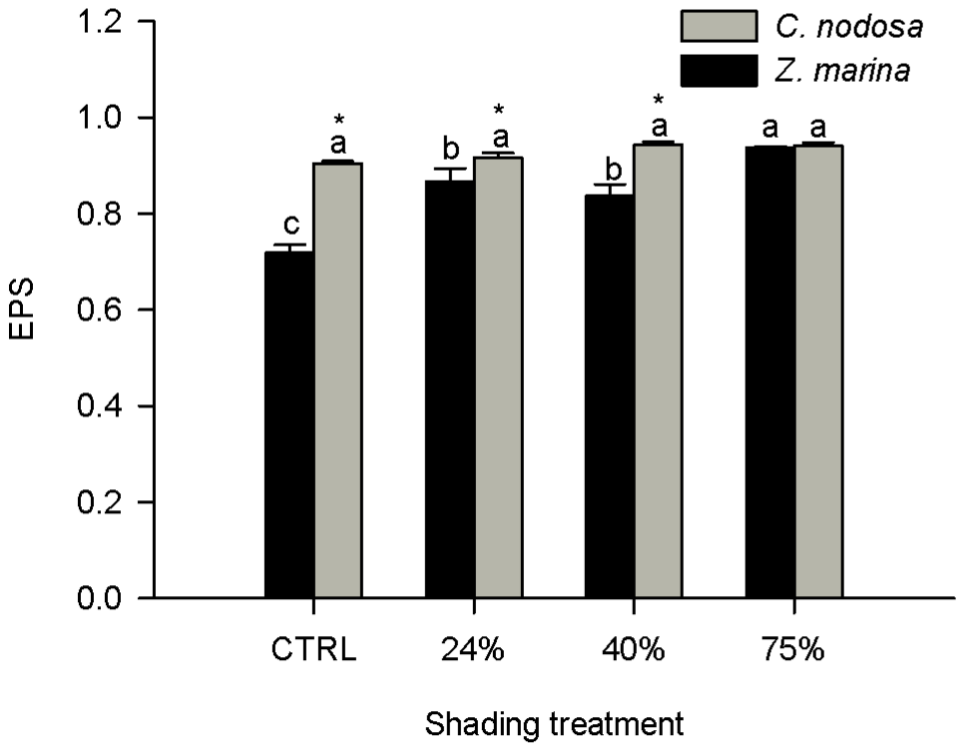
Epoxidation state of xanthophyll cycle pigments [EPS  =  (V + 0.5A)/(V+A+Z)] in *Zostera marina* and *Cymodocea nodosa*. Plants submitted to shading treatments of 24, 40 and 75% of naturally available photosynthetically active radiation (CTRL). Different letters indicate significant differences between treatments, * indicates differences between species (n = 5, *p*<0.05).

The epoxidation state (EPS) of Z. marina was lower than that of C. nodosa in control plants and in those submitted to the two lowest shading levels, being identical at the 75% treatment (Fig. 4). The EPS index of Z. marina increased significantly at all levels, whereas in C. nodosa it did not respond to the shading treatment.

Table 2 summarizes total chlorophyll contents and pigment ratios that were not significantly affected by shading for both species. Significant interspecific differences are indicated. The average total chlorophyll content was nearly two-fold higher in C. nodosa and the ratios of lutein epoxide per total chlorophyll and per total VAZ pool were also 3.4 and 6.6 times higher, respectively, than in Z. marina. The ratio of total VAZ per total chlorophyll was two-fold higher in the later species.

**Table 2:** Photosynthetic pigment contents and ratios in leaves of *Zostera marina* and *Cymodocea nodosa*.

	*Z. marina*	*C. nodosa*
Chl T (µmol g DW^−1^)	2.31±0.21	4.41*±0.34
Chl *a*/*b* (µmol/µmol)	2.30±0.05	2.42*±0.03
L/Chl T (mmol/mol)	121.90±8.85	125.70±11.71
Lx/Chl T (mmol/mol)	2.03±0.37	6.95*±0.75
Lx/VAZ (mmol/mol)	0.024±0.004	0.160*±0.011
VAZ/Chl T (mmol/mol)	88.2±6.5	42.3*±3.0
β-car/Chl T (mmol/mol)	107.6±7.4	92.3±8.3

Values are means ± se (n = 20, p<0.001). Chl T  =  total chlorophyll, Chl *a*/*b*  =  chlorophyll *a* to chlorophyll *b* ratio, L  =  lutein, Lx  =  lutein epoxide, VAZ  =  sum of violaxanthin, antheraxanthin and zeaxanthin, β-car  =  β-carotene.

*indicates significant differences between species.

### Non-structural carbohydrates

**Figure 5:**
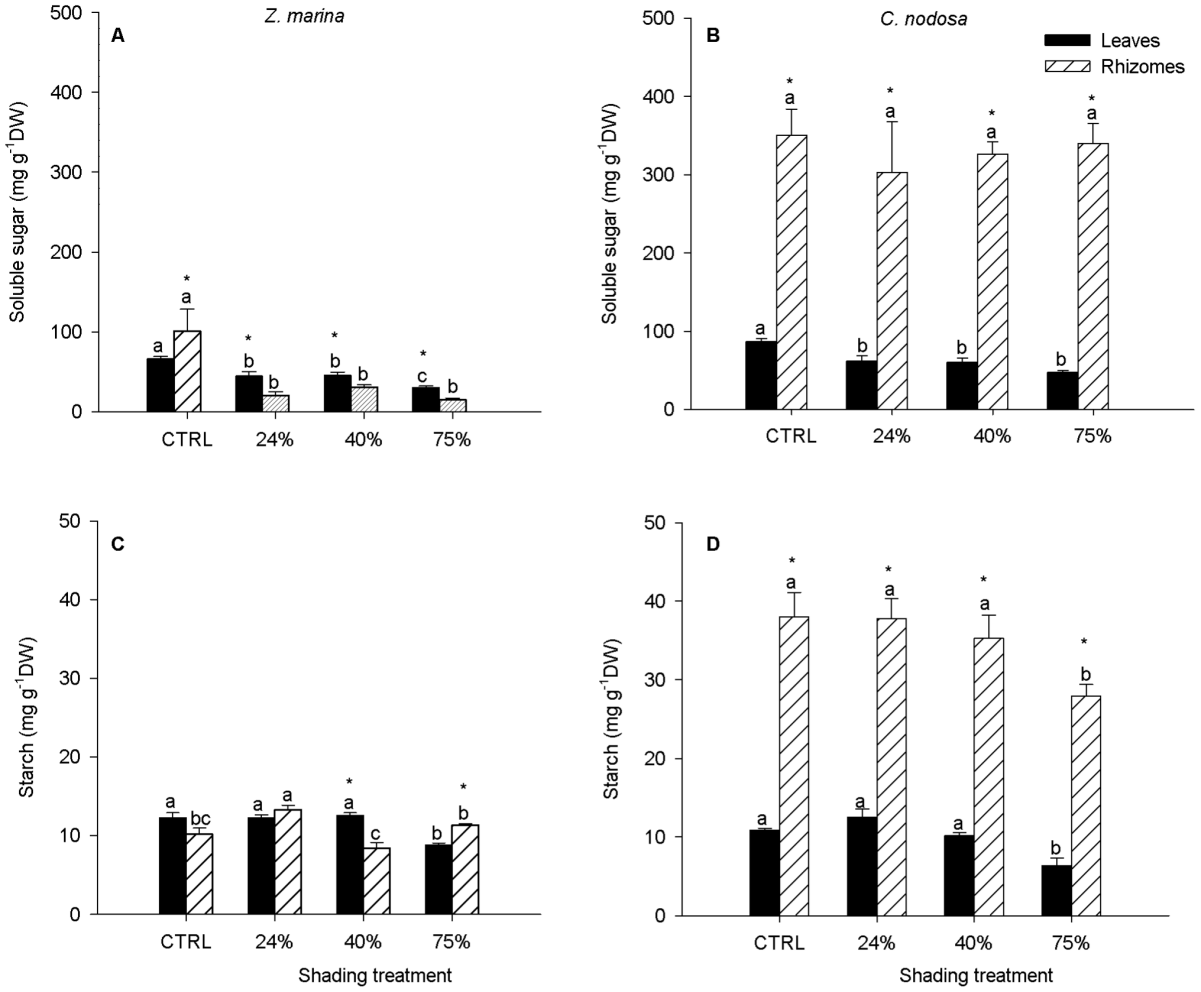
Soluble sugars (A and B) and starch (C and D) content in leaves and rhizomes of *Zostera marina* (A, C) and *Cymodocea nodosa* (B, D). Plants submitted to shading treatments of 24, 40 and 75% of naturally available photosynthetically active radiation (CTRL). Different letters indicate significant differences between treatments, * indicates differences between leaves and rhizomes (n = 5, *p*<0.05).

*Z. marina* and *C. nodosa* control plants presented an identical level of soluble sugars in the leaves and this level declined significantly with shading in both species (Fig. 5 A and B). However, *C. nodosa* control plants had ca. 3.5 fold more soluble sugar stored in the rhizomes than *Z. marina* and this high level was not affected by shading. The rhizome sugar content of *Z. marina* decreased between 70 and 85% in relation to control. In addition, *Z. marina* and *C. nodosa* control leaves showed identical starch contents (Fig. 5 C and D), and in both species a decrease was only observed under the highest shading level. In contrast, *C. nodosa* control plants had nearly four-fold more starch stored in the rhizomes than *Z. marina*, showing only a significant decrease at the highest shading level.

### MDA and phenols

**Figure 6:**
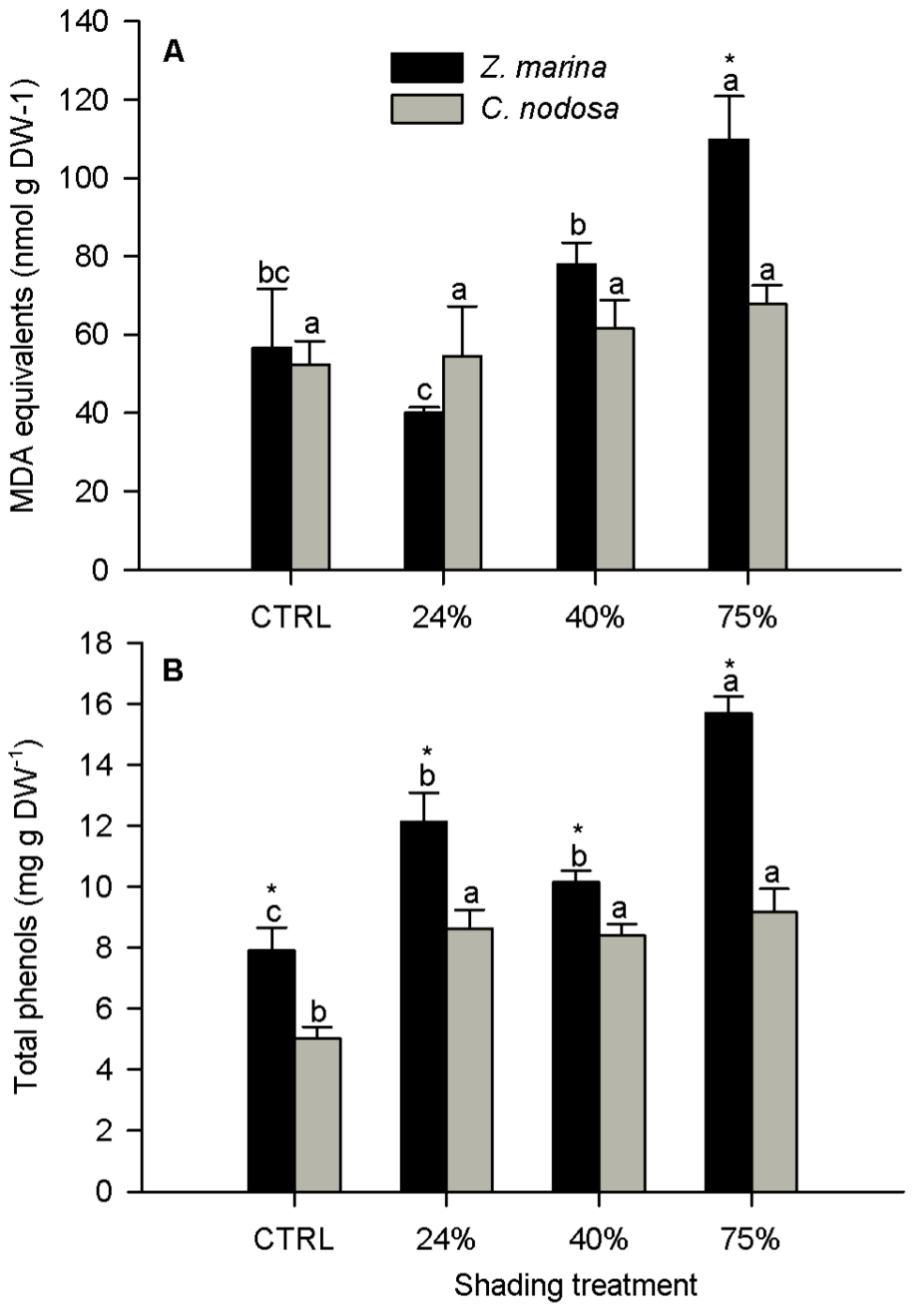
Malondialdehyde (MDA, A) and total phenols (B) in leaves of *Zostera marina* and *Cymodocea nodosa*. Plants submitted to shading treatments of 24, 40 and 75% of naturally available photosynthetically active radiation (CTRL). Different letters indicate significant differences between treatments, * indicates differences between species (n = 5, *p*<0.05).

MDA foliar content was not significantly affected by shading in *C. nodosa* whereas in *Z. marina* it showed a significant increase only at 75% shading (Fig. 6 A). MDA values were always similar in both species except at 75% shading. Total phenols increased with shading in both species (Fig. 6 B). *Z. marina* plants always had significantly higher phenol content than *C. nodosa*.

## Discussion

Shading induced significant alterations in the photosynthetic apparatus of both *Zostera marina* and *Cymodocea nodosa*, as revealed by the decrease of their photosynthetic activity (Fig. 1 and Table 1). Both species showed a continuous, yet distinct, photoacclimatory response. In *Z. marina* the maximum photosynthetic rate (*P_m_*) decreased with the shade levels, revealing a decreased capacity to photosynthesize as well as a lower photosynthetic efficiency, expressed by a reduction in the ascending slope of the light-limited part of the P-E curve (α). As *P_m_* and α decreased proportionally, the saturation irradiance (*I_k_*) didn't change significantly with the shading levels, In contrast, the decrease of *P_m_* in *C. nodosa* was accompanied by a significant increase of the photosynthetic efficiency at low light intensities (α, resulting in a decrease (to less than 20%) in *I_k_*. The higher photosynthetic efficiency of *C. nodosa* at low light intensities may even be amplified by its higher content on total chlorophyll (Table 2), supporting the hypothesis that *C. nodosa* is generally more able to deal with low light conditions than *Z. marina*.

Both *C. nodosa* and *Z. marina* presented foliar chl *a*/*b* ratios below 2.5 (Table 2), values frequently attributed to shade leaves in terrestrial plants [29]. Additionally, *C. nodosa* displayed higher Lx/ChlT and Lx/VAZ ratios and lower VAZ/ChlT and β-car/ChlT ratios than *Z. marina*. This is typical of shade acclimated leaves [30] and points to a higher light harvesting efficiency, advantageous in a low light environment where less excitation energy reaches the reaction centres, which become underused unless the light capture capacity is enhanced. However, in an apparent contradiction, *C. nodosa* control plants presented significantly lower photosynthetic efficiency than *Z. marina.* This might be explained by potentially higher rates of oxygen consuming processes such as photorespiration, Mehler reaction, cellular respiration, chlororespiration and mitochondrial alternative oxidase pathway [31], [32], [33], [34] in non-shaded *C. nodosa* plants. Some of these processes, namely photorespiration, Mehler reaction and cellular respiration, also produce reactive oxygen species (ROS) [35], which induce the peroxidation of cellular membrane lipids and lead to the production of malondialdehyde (MDA) [36]. Thus, higher rates of those metabolic pathways should be reflected on higher MDA values in *C. nodosa*, particularly in control plants. As well, assuming that the rate of those metabolic pathways would decrease with shade, so would the MDA content. This decrease of oxygen consuming processes with shade would be in line with the increase of the photosynthetic efficiency (α). However, there were no differences on the MDA content of *C. nodosa* plants under the different shading treatments (Fig. 6 A), meaning that either there was no change on the amount of ROS production or, most likely, there was an efficient ROS scavenging machinery operating in control plants and keeping ROS below the limit from which they cause significant oxidative stress. In *Z. marina* the intensification of shading was followed by the increment on oxidative stress, which reached its maximum at 75% shading level (Fig. 6 A). In this species the increment on oxidative stress was accompanied by a significant decrease on photosynthetic efficiency (α, which could be related to the up regulation of O_2_ consuming biochemical pathways. Unlike *C. nodosa*, *Z. marina* is reported to have photorespiration [37], but the up regulation of photorespiration, Mehler reaction and chlororespiration is commonly related with high light intensities and/or temperature stress, but not with shading. Nonetheless, an increment on oxidative stress in the aquatic macrophyte *Potamogeton crispus* was attributed to the unbalance of C-N metabolism under low light [38]. This kind of mechanism could be simultaneously responsible for the increase of oxidative stress in *Z. marina*, and for the maintenance of the MDA levels in *C. nodosa*. Plant phenolic compounds are carbon based and are believed to act as antioxidants [39] [40]. The likely decrease in O_2_-consuming metabolic pathways together with the increase on leaf phenols seems to have contributed to the maintenance of MDA concentrations in shaded *C. nodosa* plants. In *Z. marina*, the increase on total phenols was not enough to prevent oxidative stress, which in turn may be related to the decrease of α with shading, since ROS are known to decrease the rate of repair of photosystem II [41]. Leaf total phenols were always higher in *Z. marina* then in *C. nodosa*, probably due to constitutive differences between the two species.

While *C. nodosa* displayed a pigment content typical of shade acclimated leaves [30], regardless of the shading level, the photosynthetic apparatus of *Z. marina* tended to acclimate as shading levels increased. Nevertheless it was only at the 75% shading level that significant increases of the neoxanthin, lutein pool, violaxanthin and β-carotene foliar contents (Figs. 2 and 3) were detected in *Z. marina*. Although most pigment content and ratios were higher in *C. nodosa* regardless of light treatment (Table 2 and Fig. 3), this difference was attenuated by the increment on *Z. marina* photosynthetic pigments as shade treatment increased (Fig. 3). The foliar concentrations of β-carotene and of the xanthophylls neoxanthin, lutein epoxide plus lutein (LxL), and violaxanthin were always significantly higher in control *C. nodosa* plants (Fig. 3), being neoxanthin and LxL foliar contents similar to the median values found in several shade leaves of different species [30]. The xanthophylls violaxanthin, neoxanthin and Lx have been associated to a more stable and efficient structure of the LHCII, thus contributing to more efficient light harvesting and transmission of excitation energy to chlorophyll *a* [9] [10] [42] [43] [44]. β-carotene is a precursor of xanthophylls [45] and its increment in *Z. marina* leaves in response to the highest level of shading might be related with the need for neoxanthin and violaxanthin synthesis. On the other hand the difference in the LxL content between the two species resulted mainly from the difference in the lutein epoxide (Lx) content, which was significantly higher in *C. nodosa*, both on a total chlorophyll and on a xanthophyll cycle pigments basis (data not shown). The epoxidation state (EPS  =  (V + 0.5A)/VAZ) reflects the proportion of the VAZ cycle pigments that resulted from the epoxidation of zeaxanthin [21]. Shading did not induce any alteration in *C. nodosa* EPS but this index increased significantly in *Z. marina* (Fig. 4), mainly due to the significant increase in violaxanthin. At the 75% shading level, the foliar concentration of violaxanthin was significantly higher in *Z. marina* than in *C. nodosa*, whose violaxanthin levels were unaffected by the shading treatments. These pigment data indicate that *C. nodosa* has a constitutively higher efficiency on light processing at the antennae level when compared to *Z. marina*, which changed its pigment contents to acquire a better capacity to use light as shading increased.

The epoxidation of zeaxanthin to violaxanthin is an O_2_ and energy consuming process [46]. Thus the epoxidation of zeaxanthin to violaxanthin may also contribute to lower photosynthetic efficiency and competes with other metabolic processes for energy, adding yet another disadvantage for *Z. marina* under low light conditions.

*Zostera marina* is a shallow growing species, most likely due to its relatively high light requirement [23]. In Ria Formosa, these plants grow in their southern distribution limit in Europe, where the high summer temperatures lead to increased respiratory rates and the plant carbon balance may be negative, as is the case in the east American coast [47]. On the other hand, winter conditions of increased turbidity reduce the available light and require an efficient photoacclimation as a condition to maintain a positive carbon balance. *Z. marina* has been previously reported as being able to photoacclimate to low irradiance levels under summer conditions [48] and in a much lesser degree also during winter periods, where it is much more vulnerable [49]. In this study, whereas some photoacclimation effort was evidenced, namely in the adjustment of the photosynthetic rates and the pigments pool, it appears that the crucial factor playing against *Z. marina* was its carbon allocation strategy.Under reduced light conditions, seagrasses mobilize stored carbohydrates to maintain metabolic processes [7]. Our data show that *Z. marina*, while normally maintaining leaf soluble sugar levels identical to *C. nodosa*, had ca. 3.5 fold less sugars stored in the rhizomes (control plants data). Furthermore, while [50], [23] reported higher (up to 10 times) soluble sugar contents in the rhizomes than in the leaves of *Z. marina*, in our study rhizomes had only ca. 1.6 times more sugar than leaves, in control plants. After shading, sugar levels were always significantly lower in rhizomes than in leaves, indicating a severe degradation of the energy storage conditions. Finally, the absolute values of soluble sugars determined in this study for both leaves and rhizomes were 4 to 6 times lower than those found in Californian populations of *Z. marina* by [49]. Similarly lower values were reported by [23] for plants collected in the southern distribution limit of this species in the east American coast, which corroborates the idea that *Z. marina*'s apparent limited flexibility to allocate and use carbohydrate reserves is greatly evidenced closer to its southern distribution limits. Relatively low starch values also appear to be characteristic of this species [47], [23], probably as means of saving inter-conversion energy and maintaining a more readily available energy source. The fact is that in this study no rhizome starch mobilization was observed after the shading treatment. On the other hand, *C. nodosa* showed only some decrease in the leaf sugar content in response to shading and the rhizome pool was not affected. The leaf and rhizome starch contents only declined following the most severe level of the shading treatment.

The different carbohydrate energy storage strategies shown between *C. nodosa* and *Z. marina* clearly favour *C. nodosa*'s resilience to light deprivation. This *C. nodosa*'s carbohydrate storage strategy is also likely to be beneficial in response to other environmental disturbances, besides being effective in coping with light reduction. For example, it has been shown that *C. nodosa* is highly resilient to disturbances such as burial and transplanting [51], [52], [53].

Although the effects of the different shading levels are noticeable in some aspects, what emerges as the most striking outcome of this experiment is the remarkable difference between the strategies adopted by the two species in dealing with a short-term decline in light availability. This difference configures *C. nodosa* as a more resilient species to transient light attenuation periods, mostly due to its constitutive arrangement of the pigment pool and to its carbohydrate storage and allocation strategy. On the other hand, *Z. marina* revealed a lower tolerance to light reduction, mostly due to a higher energy-requiring re-arrangement of the pigment pool under low-light conditions and also to a less effective strategy of carbohydrate storage and use.

The results suggest that *Z. marina* is close to a light mediated ecophysiological threshold in Ria Formosa, with only a short margin to deal with transient changes in light availability, which are common in a costal system such as Ria Formosa lagoon. Thus, potentially increasing disturbances in the light environment of the lagoon can only further contribute to its decline. On the other hand, our results also indicate that this kind of experimental approach can be a useful tool to investigate interspecific competition processes from the ecophysiological point of view, particularly as it allows some degree of trend prediction based on the specific photophysiological characteristics and acclimation potential of target species.
